# Greenhouse gas emissions from the water–air interface of a grassland river: a case study of the Xilin River

**DOI:** 10.1038/s41598-021-81658-x

**Published:** 2021-01-29

**Authors:** Xue Hao, Yu Ruihong, Zhang Zhuangzhuang, Qi Zhen, Lu Xixi, Liu Tingxi, Gao Ruizhong

**Affiliations:** 1 Inner Mongolia Key Laboratory of River and Lake Ecology, School of Ecology and Environment, Inner Mongolia University, Hohhot, China; 2 Key Laboratory of Mongolian Plateau Ecology and Resource Utilization Ministry of Education, Hohhot, China; 3 Department of Geography, National University of Singapore, Lower Kent Ridge Road, Singapore, Singapore; 4 Inner Mongolia Water Resource Protection and Utilization Key Laboratory, Water Conservancy and Civil Engineering College, Inner Mongolia Agricultural University, Hohhot, China

**Keywords:** Carbon cycle, Climate change

## Abstract

Greenhouse gas (GHG) emissions from rivers and lakes have been shown to significantly contribute to global carbon and nitrogen cycling. In spatiotemporal-variable and human-impacted rivers in the grassland region, simultaneous carbon dioxide, methane and nitrous oxide emissions and their relationships under the different land use types are poorly documented. This research estimated greenhouse gas (CO_2_, CH_4_, N_2_O) emissions in the Xilin River of Inner Mongolia of China using direct measurements from 18 field campaigns under seven land use type (such as swamp, sand land, grassland, pond, reservoir, lake, waste water) conducted in 2018. The results showed that CO_2_ emissions were higher in June and August, mainly affected by pH and DO. Emissions of CH_4_ and N_2_O were higher in October, which were influenced by TN and TP. According to global warming potential, CO_2_ emissions accounted for 63.35% of the three GHG emissions, and CH_4_ and N_2_O emissions accounted for 35.98% and 0.66% in the Xilin river, respectively. Under the influence of different degrees of human-impact, the amount of CO_2_ emissions in the sand land type was very high, however, CH_4_ emissions and N_2_O emissions were very high in the artificial pond and the wastewater, respectively. For natural river, the greenhouse gas emissions from the reservoir and sand land were both low. The Xilin river was observed to be a source of carbon dioxide and methane, and the lake was a sink for nitrous oxide.

## Introduction

As the concentration of greenhouse gas increases, the global warming effect becomes more pronounced^1^. Carbon dioxide (CO_2_), methane (CH_4_) and nitrous oxide (N_2_O) have been shown to dominate the well-mixed greenhouse gas (GHG), contributing 80% of the positive radiative forcing that drives climate change^2,3^. CO_2_ has long been known as an important greenhouse gas, and CH_4_ is also an important greenhouse gas. The global warming potential of CH_4_ in 100 years is 25 times that of CO_2_, and the contribution rate to the greenhouse effect is approximately 22%^4,5^. The N_2_O molecule is a powerful greenhouse gas that has a global warming potential 296 times greater than that of CO_2_^6^. Global warming could have a significant impact on local and regional climatic regimes, which would in turn impact hydrological and water resources systems^1,7^. The terrestrial ecosystem carbon cycle and its driving mechanisms are important components of current global change research. They are the key to predicting future atmospheric CO_2_ changes and global warming. The carbon cycle of the terrestrial ecosystem is mainly reflected in the exchange of CO_2_ on land and in lakes, rivers and the atmosphere, as well as in the direct transport of carbon to the ocean by river action^8,9^.

The river system connects the two carbon banks of land and sea. It is a key link in the global carbon cycle and the main channel for land-based carbonaceous materials to enter the sea^10^. The river carbon cycle refers to the entire process of carbon sources from different sources in the terrestrial system entering the river network system in a variety of forms under the influence of machinery, biochemistry and human activities. Rivers are significant source of greenhouse gas emissions. It is estimated that aquatic systems contribute more than 50% to atmospheric CH_4_, and global river N_2_O emissions have gradually exceeded 10% of human emissions^11,12^. The greenhouse gas emissions of urban rivers are more significant compared with natural rivers. The N_2_O, CO_2_ and CH_4_ escaping from rivers are mainly derived from microbial processes such as microbial degradation, acetic acid fermentation, ammonia oxidation and the denitrification of sediments^13,14^. As a reaction matrix, the increase in soluble inorganic nitrogen and soluble organic carbon stimulates microbial activity in the aquatic environment and promotes CO_2_, CH_4_ and N_2_O production^15,16^.

Inland waters (streams, rivers, lakes and reservoirs) have been gradually recognized as important sources of greenhouse gas release into the atmosphere^17^. Many regional studies on inland waters have proposed a specific focus on the emissions of CO_2_, CH_4_, and N_2_O^18^. However, only a few studies have assessed the three GHG concentrations together in a river system. Most of the research on greenhouse gas emissions in grassland areas has focused on soil systems but rarely on inland river systems^19^. The transverse carbon and nitrogen cycle of an inland river is generated along with the direction of the river but disappears into the terrestrial cycle with the river. The longitudinal cycle of carbon and nitrogen is exchanged by the water–air interface. Differently from a fresh water river connecting the land and the ocean, the carbon and nitrogen cycle of an inland river does not enter the ocean system but directly enters the land system in a short time.

The Xilin River basin is located in the inland river basin of arid and semiarid steppe areas. The Xilin River is a seasonal river with a low network density, highly meandering and no obvious riverbed in the downstream, and ends with a terminal lake. The grassland regions are affected by different degrees of human activities. The greenhouse gas emissions of grassland rivers under the influence of different human activities have rarely been studied and our study intended to understand the carbon emission mechanism of rivers in grassland region and provide a reference for the greenhouse gas emissions of global grassland rivers. The specific purposes of this study were to (1) explore the spatial and temporal variations of greenhouse gas emissions at the water–air interface; (2) explore effects of land use on emissions of greenhouse gas and (3) analyze the effects of human activities on emissions of greenhouse gas.

## Materials and methods

### Study sites

**Figure 1:**
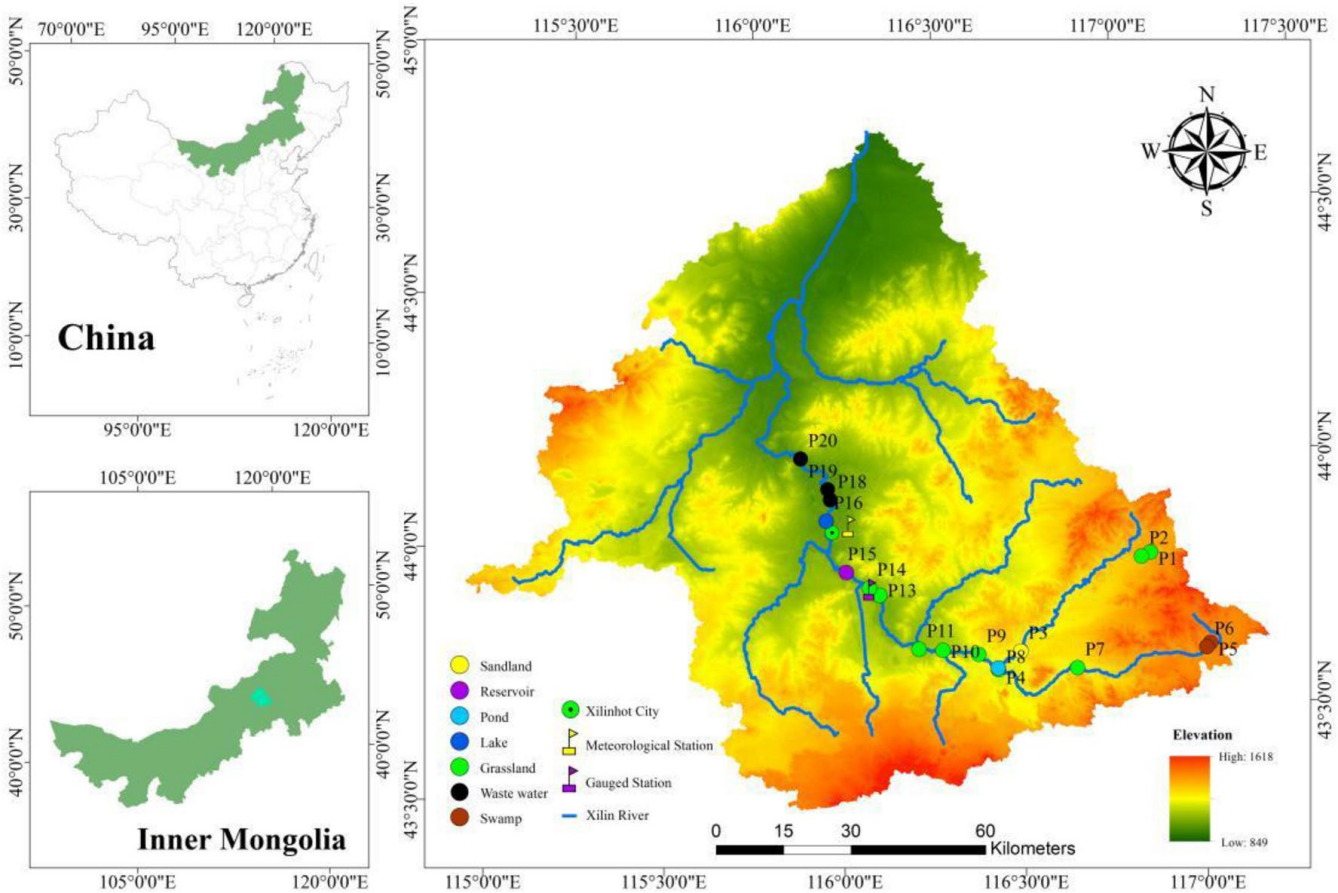
Xilin River Basin and sampling sites (generated by Arcgis10).

The Xilin River Basin is in the southeastern part of the Inner Mongolia Autonomous Region in China (E115° 00′ ~ 117° 30′ and N43° 26′ ~ 44° 39′) (Fig. 1). It is located at the western extension of the lower hills and hills of the greater Xingan mountains in the middle and eastern part of the Inner Mongolia plateau. In the north, it is characterized by an alternating distribution of low mountains and hills and high plains, and in the south, it is a multistage basalt platform. The middle area of these two terrains is mainly sandy dunes, and the terrain gradually declines from the east to the west^20^. The Xilin River Basin covers an area of 10,542 km^2^, and the average altitude is 988.5 m. The total length of the Xilin River is 268.1 km, with an average channel drop of 1.25%; however, it is cut off nearly 124.7 km below the Xilin Reservoir^21^. The Xilin River Basin is dominated by grasslands, followed by swamps, sand land and urban land^22^. The grassland area of the Xilin River Basin accounts for 88.35% of the total drainage area, and the water area accounts for 0.37%. The climate type of the Xilin River Basin is a temperate semiarid continental monsoon climate with climatic characteristics, for example, of less precipitation, more evaporation and greater daily temperature difference. According to the meteorological data of the Xilinhot Meteorological Station from 1968 to 2015, the annual average precipitation was 278.9 mm, the annual average evaporation was 1862.9 mm, the annual average temperature was 2.8 °C, and the annual mean wind speed was 3.4 m s^−1^.

### Sampling procedures and analysis

This study conducted four rounds of field work in April, June, August, and October in 2018. The design of the eighteen sampling points takes into account the changes of land use types. Fourteen sites on the main stem and four sites on the tributary were selected for sampling and measurement (Fig. 1, Table 1). The types of land use on the tributary mainly included grassland and sand land. The upstream of the Xilin River is swamp. The Xilin River flows to the grassland section in the upper stream of the Xilin River Reservoir. The downstream of the Xilin River flows through the artificial lake in Xilinhot City.

**Table 1:**
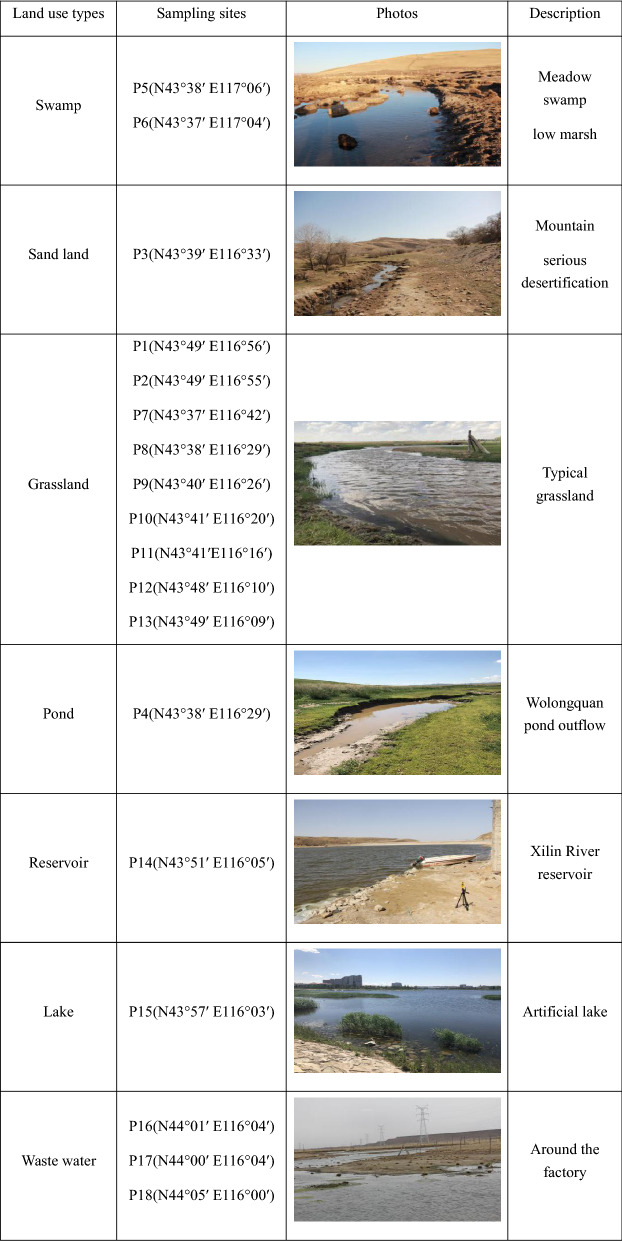
Sampling sites classified by land use type.

*These pictures were taken by myself.

The collected water samples were subjected to low-pressure suction filtration through Whatman GF/F filters (nominal pore diameter of 0.7 μm). The fiber filter was prefired in a muffle furnace at 450 °C. pH, water temperature (T_w_), salinity (Sal), dissolved oxygen (DO), and total dissolved solids (TDS) were measured by a portable water quality analyzer. pH and T_w_ were measured using a portable pH meter (WTW). Alkalinity (Alk) was titrated with 0.1 mol L^−1^ hydrochloric acid (HCl) within 10 h after sampling. HCO_3_^–^ represents 96% of the alkalinity when the pH ranges from 7 to 10^10^. Alk was determined by on-site titration. Total nitrogen (TN) was determined by the alkaline potassium persulfate digestion-UV spectrophotometric method^23^, and total phosphorus (TP) was determined by the ammonium molybdate spectrophotometric method^24^. Flow velocity of water (V_w_) was measured using a doppler portable flow meter (DPL-LS10), and the flow discharge was calculated by V_w_, river width and depth.

### GHGs measurement

#### pCO_2_, pCH_4_, and pN_2_O measurements

In this study, surface water *p*CO_2_ was calculated using the headspace equilibrium method. By using an 1100 mL conical flask, 800 mL of water was collected to the depth of 10 cm below the water surface and the remaining volume of 300 mL was filled with ambient air. The flask was immediately closed with a lid and vigorously shaken for 3 min to equilibrate the gas in the water and air. The equilibrated gas was automatically injected into the calibrated Li-7000 gas analyzer. The Li-7000 CO_2_/H_2_O analyzer was connected to a computer interface that allowed *p*CO_2_ recording for two seconds. The measurements at each site were repeated three times and the average was calculated (analytical error below 3%). The original surface water *p*CO_2_ was finally calculated by using solubility constants for CO_2_ and the headspace ratio^10^. After shaking the conical flask, the gas extracted from flask was injected into the vacuum cylinder (Labco Exetainer). *p*CO_2_, *p*CH_4_, and *p*N_2_O in the water column were measured using a gas chromatograph.

*p*CO_2_ from the water was also calculated using the CO_2_SYS program^25^, which has been widely employed for aquatic *p*CO_2_ calculations^26,27^. T_w_, Alk and pH were essential data for such calculation^28^.

**Figure 2:**
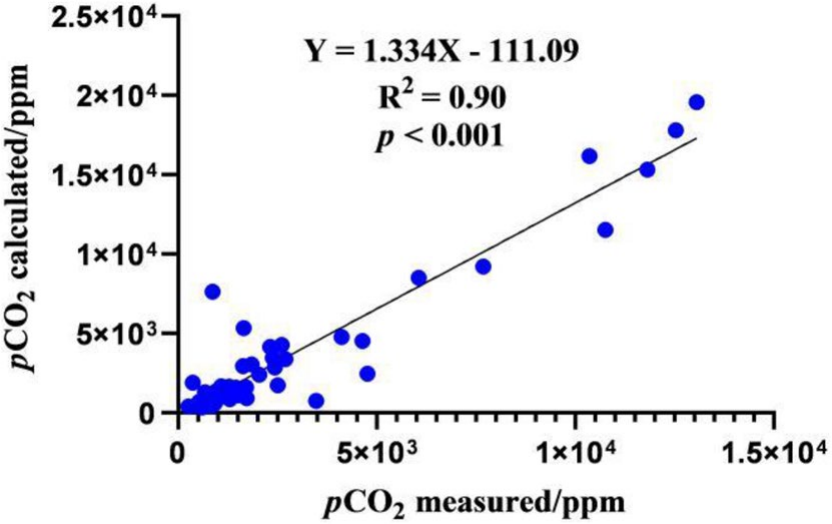
Comparison of the results of river *p*CO_2_ at all sampling sites on the Xilin River by the measured and calculated methods.

The *p*CO_2_ calculated was slightly higher than the *p*CO_2_ measured directly by gas chromatography (R^2^ = 0.90) (Fig. 2). The reason for the higher calculated *p*CO_2_ value was due to the error generated from pH and T_w_ measurements or the artificial error that occurred during the titration. The directly measured data could be used for analysis and discussion.

#### Greenhouse gas emissions calculation

In this study, *F*CO_2_ was measured by the floating chamber method and an Li-7000 CO_2_/H_2_O analyzer (Li-Cor, USA). The Li-7000 instrument was calibrated with standard CO_2_ gases of 500 ppm and 1000 ppm before each measurement. *F*CH_4_ and *F*N_2_O were measured by the floating chamber method. 60 mL of gas was taken from the floating chamber every three minutes; five samplings were taken and injected into a vacuum cylinder.

The static chamber volume was 17.8 L, and the covered water area was 0.09 m^2^. The chamber was covered with tinfoil to reduce the influence of sunlight. The temperature inside the chamber was measured with a thermometer. At the beginning of each experiment, the chamber was placed in the air near the monitoring point. The instrument automatically recorded the air CO_2_ concentration and ambient atmospheric pressure. When the chamber was placed on the water surface, the analyzer recorded the CO_2_ concentration every two seconds, and each measurement lasted for 6–10 min.

The greenhouse gas emissions from water were calculated using the following equation^29^:

$${\text{FGHG = (dpGHGs}}/{\text{dt)}}(V/{\text{ RTS)}}$$

where d*p*GHG/dt is the slope of greenhouse gas change within the chamber (Pa d^–1^; converted from μatm min^–1^), V is the chamber volume (17.8 L), R is the gas constant, T is chamber temperature (K), and S is the area of the chamber covering the water surface (0.09 m^2^).

## Results

### Physical and chemical parameters variation of the Xilin River

**Figure 3:**
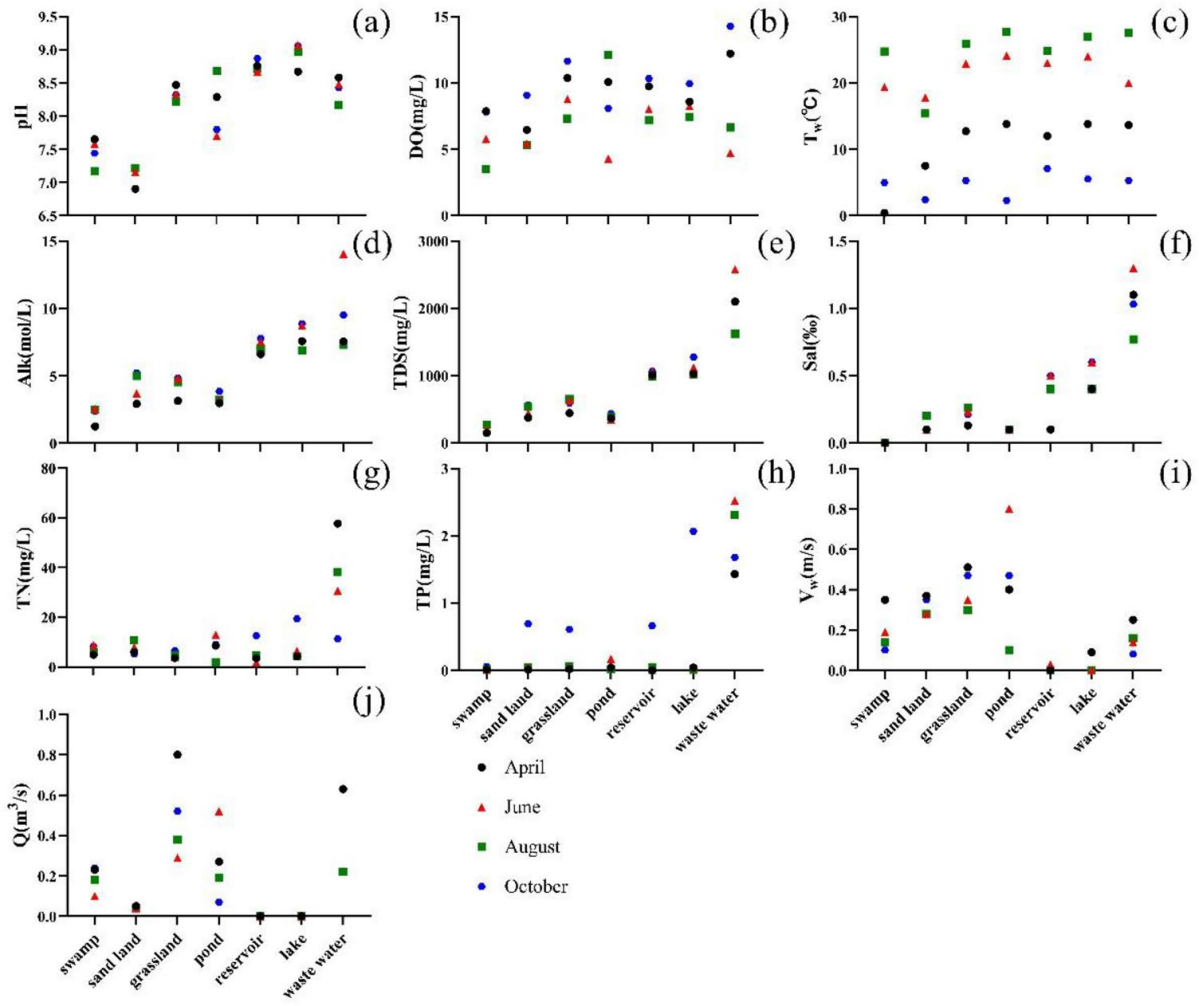
Box plot showing seasonal and spatial variation under different land use variations in six parameters of water quality(pH (**a**), DO (**b**), T_w_ (**c**), Alk (**d**), TDS (**e**), Sal (**f**), TN (**g**), TP (**h**), V_w_ (**i**), Q (**j**)).

During the sampling campaigns, pH ranged from 6.90 to 9.10, and the seasonal variation of pH was not obvious (Fig. 3a). The average annual pH was 8.20 but the spatial variation was significant. In the sand land area, the pH value was the lowest (7.12 ± 0.13). The pH value from upstream to downstream showed an overall increase trend. The concentration of DO ranged from 2.23 to 16.69 mg/L, and the average concentration of DO was 8.97 mg/L (Fig. 3b). The DO concentrations of the seasonal and spatial variables showed significant differences. In the waste water, the DO value was the highest in October and the lowest in June. The DO value in swamp and pond land use types were lower than in the other areas. T_w_ varied from 0.30 to 31.90 °C at all sampling sites, the annual mean value was 15.40 °C, and the seasonal variation of T_w_ was significant (*P* < 0.05); however, there was no significant difference in spatial distribution (*P* > 0.05) (Fig. 3c).

The Alk concentration ranged from 1.15 to 14.01 mg/L, and the average Alk concentration was 4.93 mg/L (Fig. 3d). There was no significant seasonal difference in Alk. The average value of four months was 4.31 mg/L in April, 6.04 mg/L in June, 4.91 mg/L in August, and 5.83 mg/L in October. Alk had a significant spatial change, and the Alk gradually increased from the upper reaches to the downstream. The variable range of TDS concentration was 147.00–2580.00 mg/L, and the average value was 782.00 mg/L (Fig. 3e). The seasonal variation was not significant (*P* > 0.05), but the spatial change was significant, with a significant increase trend from upstream to downstream (*P* < 0.05). Sal ranged from 0.00 to 1.30, with an average of 0.31 (Fig. 3f). From the upper reaches to the lower reaches, there was a significant increase.

TN ranged from 1.81 to 57.70 mg/L, with an average value of 10.86 mg/L (Fig. 3g). Similarly, there was a significant increase from the upper reaches to the lower reaches. TP ranged from 0.00 to 2.52 mg/L, and the average value was 0.45 mg/L (Fig. 3h). In the waste water, the TP concentration was higher than others. The variable range of V_w_ was 0.00–1.20 m/s (Fig. 3i). In the upstream, the V_w_ value was higher than that in the downstream. The Q value ranged from 0.00 to 0.80 m^3^/s (Fig. 3j). In the grassland, the Q was higher than that in other land use types.

### Seasonal variation of greenhouse gas emissions

#### CO_2_

**Figure 4:**
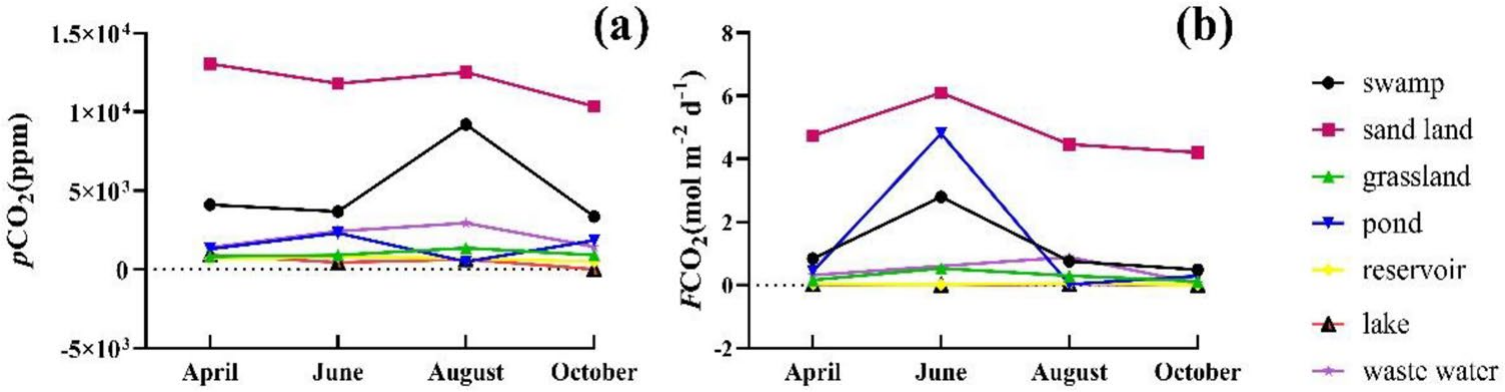
Temporal variation of *p*CO_2_ (**a**) and *F*CO_2_ (**b**) in the Xilin River in 2018.

The range of *p*CO_2_ varied from 442.54 to 13,056.85 ppm, with a four-month average of 2230.65 ppm, which was almost five times that in air (the average in the air was 402.00 ppm) (Fig. 4). To better display the spatial variation of *p*CO_2_, Fig. 4 shows the variation of *p*CO_2_ along the river. The highest value of *p*CO_2_ appeared in sand land (11,937.33 ± 1,017.37 ppm), followed by swamp (5,089.54 ± 2,397.81 ppm), wastewater (2,048.93 ± 660.43 ppm), and pond (1,486.46 ± 673.71 ppm) land uses. *p*CO_2_ in grassland, reservoir, and lake types were normally below 1000 ppm. Under different land use types, *p*CO_2_ showed different seasonal characteristics. In the swamp, grassland, and wastewater, *p*CO_2_ had the highest value in August. At all sampling sites, the average *p*CO_2_ was 1,991.77 ± 2,890.53 ppm in April, 2,247.53 ± 2,882.77 ppm in June, 2,991.71 ± 3,587.52 ppm in August, and 1,872.35 ± 2,299.81 ppm in October.

The range of *F*CO_2_ varied from 0.00 to 6.09 mol m^−2^ d^−1^, and the four-month average was 0.70 mol m^−2^ d^−1^. Figure 4 shows the spatial change of *F*CO_2_ under different land use types. The highest value of *F*CO_2_ appeared in sand land (4.88 ± 0.73 mol m^−2^ d^−1^), followed by pond (1.39 ± 1.98 mol m^−2^ d^−1^), swamp (1.22 ± 0.92 mol m^−2^ d^−1^), waste water (0.41 ± 0.35 mol m^−2^ d^−1^) and grassland (0.28 ± 0.16 mol m^−2^ d^−1^); the values of *F*CO_2_ in reservoir and lake types were within 0.02. Under the different land use types, *F*CO_2_ showed different seasonal changes. *F*CO_2_ and *p*CO_2_ showed different trends, and in sand land, swamp and grassland, the *F*CO_2_ value was highest in June. Due to the shortage of water in June, *F*CO_2_ could not be measured in the factory area. For all sampling points, the average value of *F*CO_2_ was 0.52 ± 1.06 mol m^−2^ d^−1^ in April, 1.42 ± 1.86 mol m^−2^ d^−1^ in June, 0.63 ± 1.09 mol m^−2^ d^−1^ in August, and 0.37 ± 0.95 mol m^−2^ d^−1^ in October.

The *p*CO_2_ and *F*CO_2_ values of grassland were at a low level and were only higher than those of the Xilin River Reservoir and the Xilinhot artificial lake. The *p*CO_2_ and *F*CO_2_ values of sand land were at a high level.

#### CH_4_

**Figure 5:**
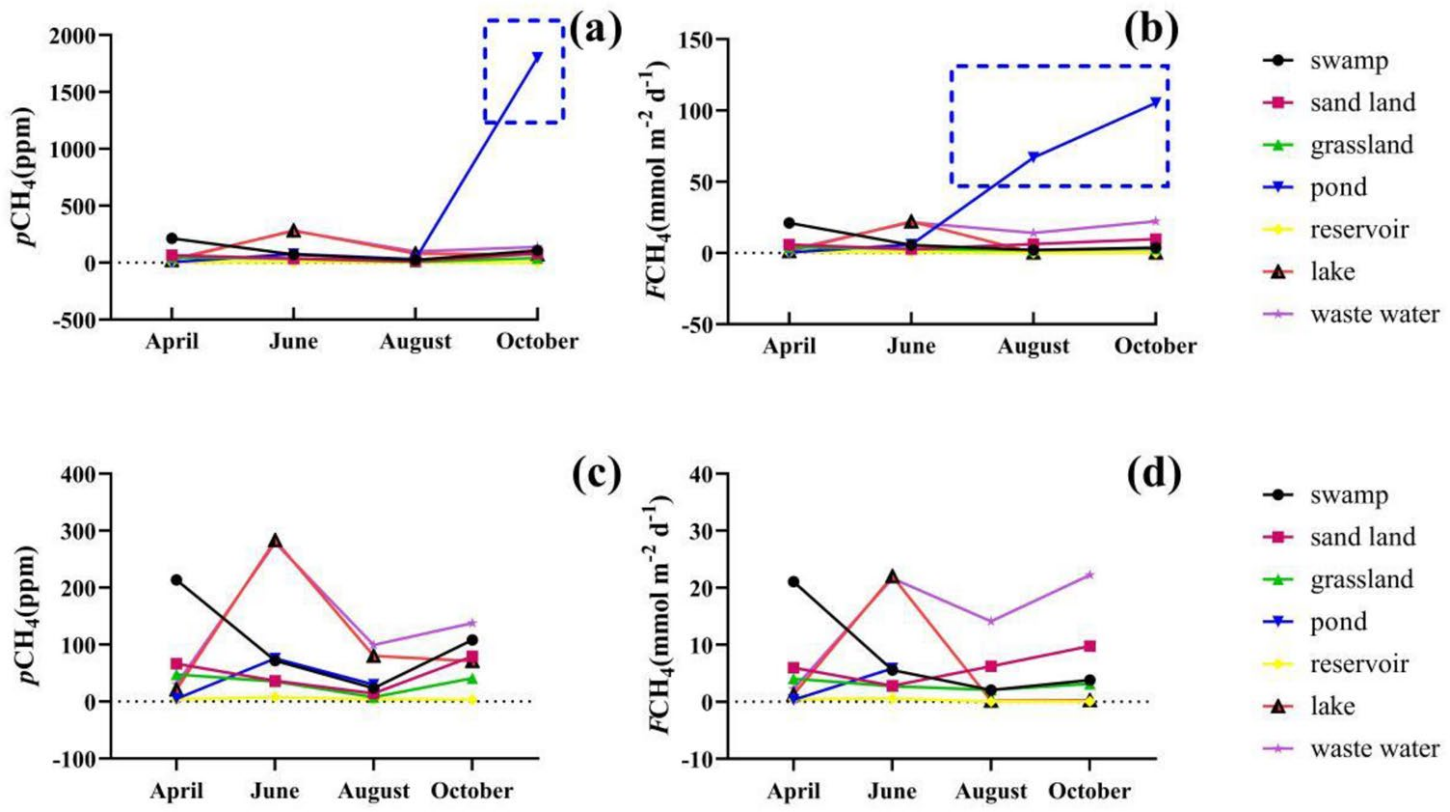
Temporal variation of *p*CH_4_ (**a**) and *F*CH_4_ (**b**) in the Xilin River in 2018 (*p*CH_4_ (**c**) and *F*CH_4_ (**d**) except those points in the blue dotted box).

The range of *p*CH_4_ varied from 2.92 to 1,800.73 ppm, with average value of 81.55 ppm. The highest value of *p*CH_4_ appeared in the pond (477.83 ± 764.20 ppm), which was much higher than the atmospheric background value, followed by waste water (136.52 ± 90.50 ppm), lake (113.86 ± 100.40 ppm), and swamp (104.26 ± 69.88 ppm), and in the remaining region *p*CH_4_ values were within 50 ppm (Fig. 5a). Under different land use types, *p*CH_4_ value showed different seasonal changes. Except for the abnormal *p*CH_4_ value of ponds in October, the value of *p*CH_4_ in June was highest affected by human activities. However, areas such as swamp, sand land, and grassland, had higher CH_4_ values in April.

The range of *F*CH_4_ was 0.06 to 105.19 mmol m^−2^ d^−1^, and the average value was 7.76 mmol m^−2^ d^−1^(Fig. 5b). The value of *F*CH_4_ was largest in the pond. There was no significant difference in the *F*CH_4_ values. Under different land use types, there were no significant seasonal differences in *F*CH_4_.

#### N_2_O

**Figure 6:**
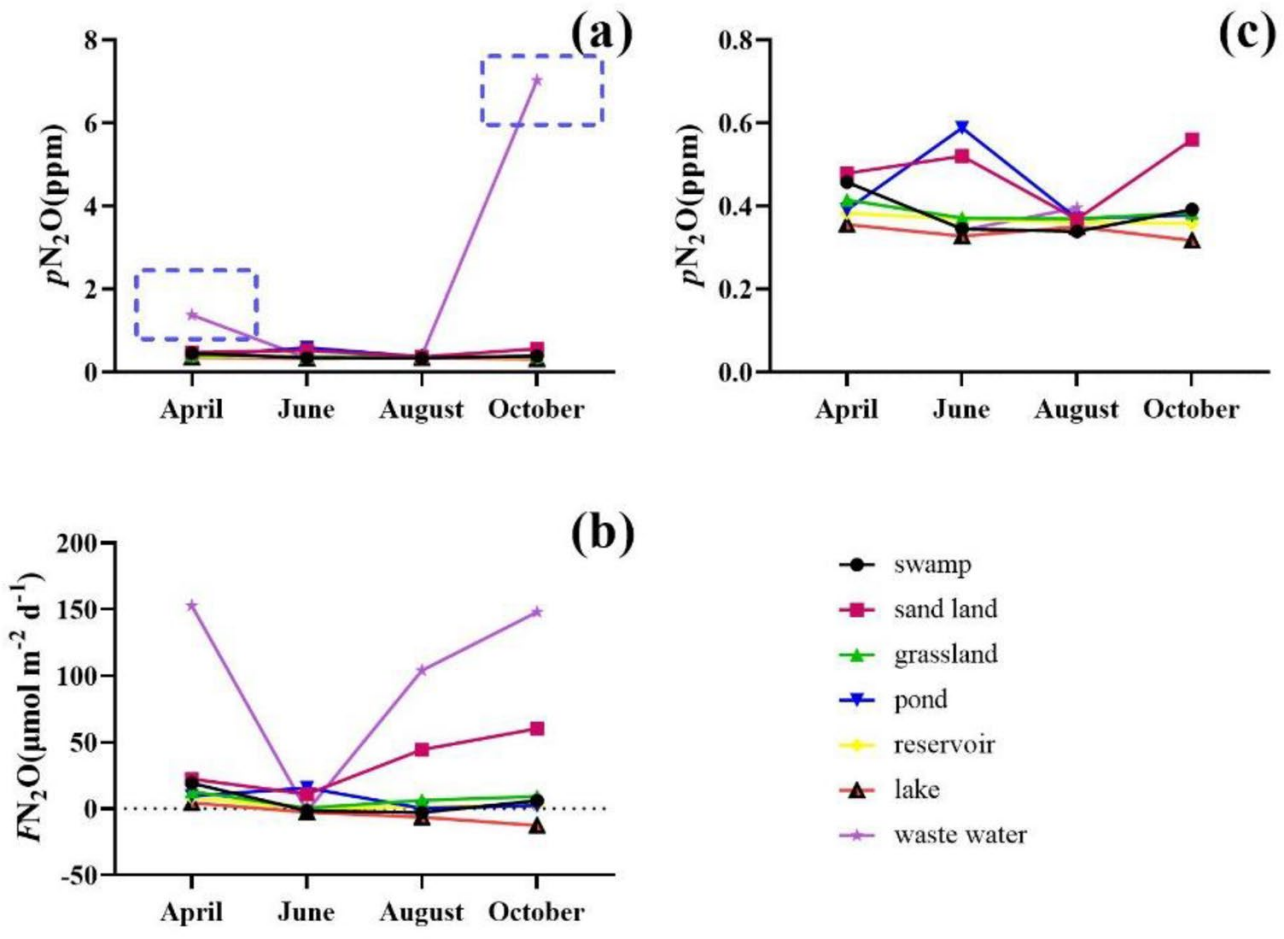
Temporal variation of *p*N_2_O (**a**) and *F*N_2_O (**b**) in the Xilin River in 2018 (*p*N_2_O (**c**) except those points in the blue dotted box).

The variation range of *p*N_2_O was 0.31–12.44 ppm, the mean value was 0.73 ppm (Fig. 6). In the factory area, the *p*N_2_O value was abnormal, and the *p*N_2_O values in April and October were higher than those in June and August. There were no obvious differences in *p*N_2_O in other areas.

The range of *F*N_2_O values was − 12.60 to 224.04 μmol m^−2^ d^−1^, and the average value was 24.32 μmol m^−2^ d^−1^. There was an** obvious spatial change in *F*N_2_O. The *F*N_2_O value of the factory area was largest, followed by sand land and grassland, yet the values of *F*N_2_O in the lake area was negative.

### Spatial variation of greenhouse gas emissions

#### CO_2_

**Figure 7:**
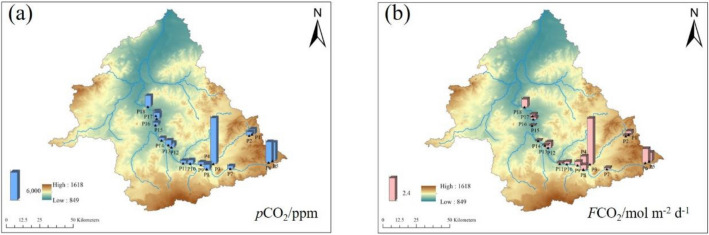
Spatial variation of *p*CO_2_ (**a**) and *F*CO_2_ (**b**) in the Xilin River in 2018 (generate by Arcgis 10).

On the main stream, the *p*CO_2_ values from upstream to downstream first decreased and then increased; they reached the lowest value in the middle stream (Fig. 7). The middle part of the tributaries was cut off, and the *p*CO_2_ value was higher at the source of the river. The spatial tendency of *F*CO_2_ value was the same as that of *p*CO_2_.

#### CH_4_

**Figure 8:**
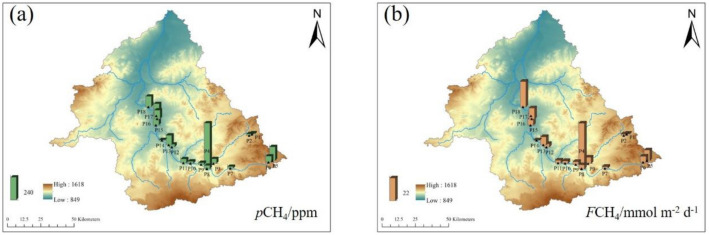
Spatial variation of *p*CH_4_(a) and *F*CH_4_(b) in the Xilin River in 2018 (generate by Arcgis 10).

On the main stream of the Xilin River, from the upstream to the downstream, the *p*CH_4_ value was higher in the river source area and increased with the flow direction of the river (Fig. 8). The value of *p*CH_4_ gradually increased in tributaries. The variation trend of *F*CH_4_ value was not obvious, and it had the lowest and negative value in the downstream of the main stream.

#### N_2_O

**Figure 9:**
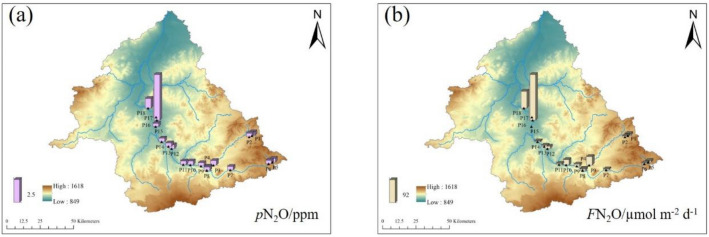
Spatial variation of *p*N_2_O (**a**) and *F*N_2_O (**b**) in the Xilin River in 2018 (generate by Arcgis 10).

The value of *p*N_2_O did not change in the tributaries but first remained stable and then increased from the upstream to the downstream of the main stream (Fig. 9). The value of *F*N_2_O fluctuated near zero and increased in the downstream area.

The global warming potential of CH_4_ is 25 times larger than that of CO_2_, and the global warming potential of N_2_O is 296 times larger than that of CO_2_^30^. For the hydrosystem of the Xilin River Basin, CO_2_ emissions accounted for 63.35% of the three GHG emissions, whereas CH_4_ and N_2_O emissions accounted for 35.98% and 0.66%, respectively.

In the swamp area, CO_2_ emissions accounted for 20.88% of the emissions of the Xilin river, and CH_4_ accounted for 6.14% (Table 2). In sand land, CO_2_ emissions accounted for 8.03% of the emission in the Xilin river, and CH_4_ and N_2_O emissions accounted for 0.45% and 0.02%, respectively. In the pond type, CO_2_ emissions accounted for 9.52% in the Xilin river, and CH_4_ and N_2_O emissions accounted for 13.46% and 0.01%, respectively. In waste water, CO_2_ emissions accounted for 7.08% of the Xilin river, and CH_4_ emission accounted for 10.38%. The hydrosystem of the Xilin River Basin showed as a source of carbon dioxide and methane; at the same time, the nitrous oxide in the lake region showed as a sink.

**Table 2:** Emissions of greenhouse gas in the Xilin river under different land use types.

	*F*CO_2_ (kg a^−1^)	%	*F*CH_4_ (kg a^−1^)	%	*F*N_2_O (g a^−1^)	%	GHG-CO_2_-eq (t a^−1^)
Swamp	542.65	20.88	1.32	6.14	2.34	0.02	0.63
sand land	208.57	8.03	0.10	0.45	1.48	0.02	0.22
grassland	1233.25	47.46	4.87	22.69	33.36	0.31	1.58
Pond	247.43	9.52	2.89	13.46	1.24	0.01	0.45
reservoir	108.07	4.16	0.44	2.07	13.95	0.13	0.14
Lake	74.65	2.87	9.62	44.82	− 19.23	–	0.73
waste water	183.91	7.08	2.23	10.38	54.06	0.51	0.35
Total	2598.55	63.35	21.47	35.98	106.43	0.66	4.10

## Discussion

### Impacts of water quality parameters on GHG

**Figure 10:**
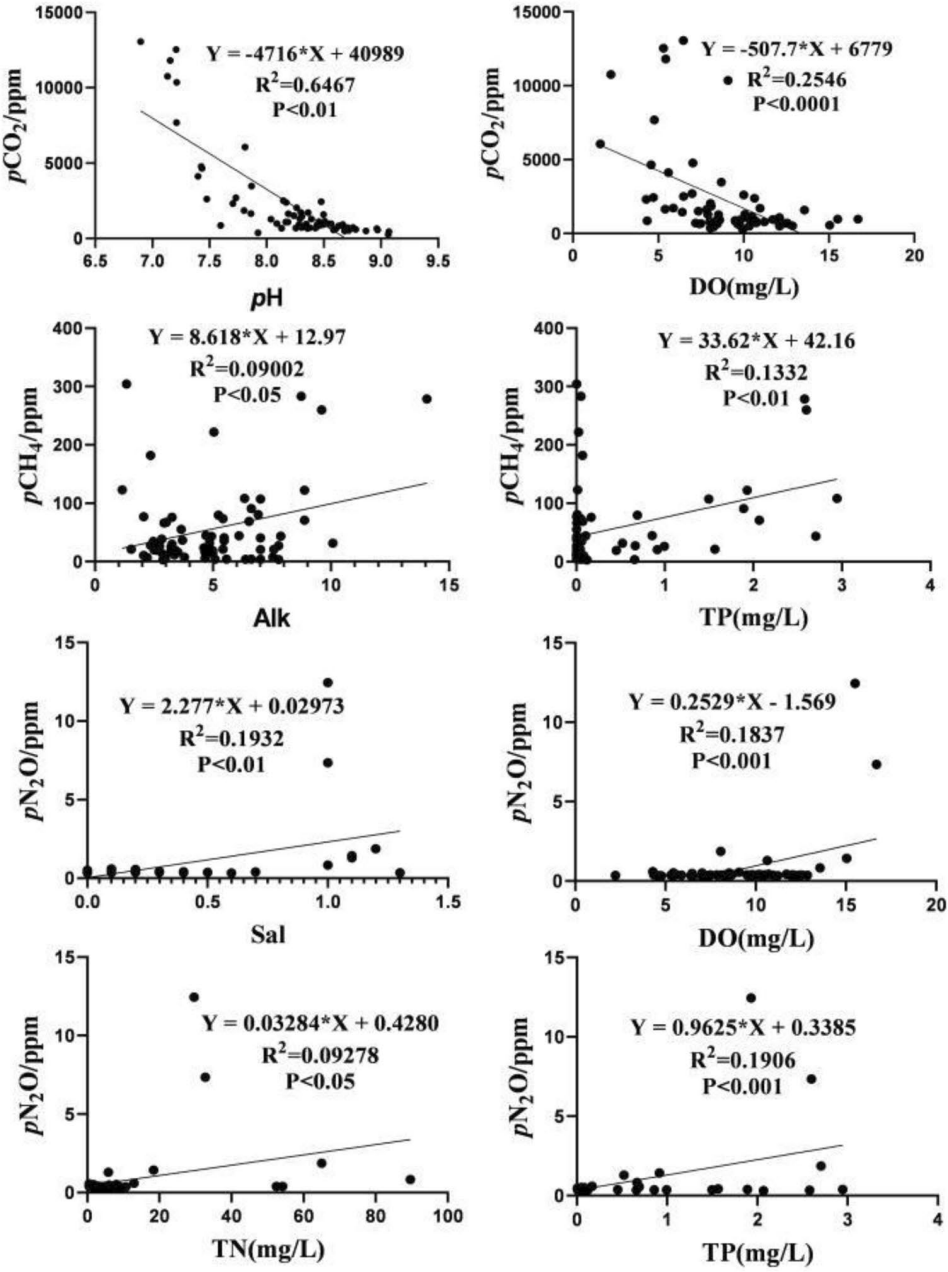
Relationship between *p*GHG and water quality parameters.

Pearson’s correlation analysis was used to analyze the correlation between eight water chemical factors with *p*GHG and *F*GHG (Fig. 10).

The main influencing factors of *p*CO_2_ were pH and DO, and *p*CO_2_ was negatively correlated with Alk, Tw, Sal, TDS, pH and DO. There was a significant negative correlation with pH (R = − 0.804, *P* < 0.01) and DO (R = − 0.505, *P* < 0.01). *p*CO_2_ was positively correlated with TN, TP and V_w_. The main influence factors of *F*CO_2_ and *p*CO_2_ were the same; however, the flow velocity (V_w_) had positive correlation with *F*CO_2_, Q (R = − 0.274, *P* < 0.05) had a significant negative correlation with *F*CO_2_. *p*CH_4_ had a significant correlation with TP (R = 0.365, *P* < 0.01). *F*CH_4_ had no significant correlation with all factors. The main influencing factors of *p*N_2_O were DO (R = 0.429, *P* < 0.01) and TP (R = 0.437, *P* < 0.01). *p*N_2_O was negatively correlated with T_w_. There was a positive correlation of *F*N_2_O with below factors: the most important were with Sal (R = 0.661, *P* < 0.01), Alk (R = 0.374, *P* < 0.01), TDS (R = 0.639, *P* < 0.01), TP (R = 0.696, *P* < 0.01), TN (R = 0.589, *P* < 0.01) and DO (R = 0.361, *P* < 0.01), which had a significant positive correlation with *F*N_2_O.

Previous studies have shown that water temperature is one of the factors that affect river *p*CO_2_ and *F*CO_2_ because the solubility of CO_2_ decreases with the rise of temperature; this has been found in many river studies around the world^31,32^. Other studies have also found that the photosynthesis of plankton has a great influence on the changes of *p*CO_2_ and *F*CO_2_ in rivers^33^. In this study, the water temperatures in June and August are higher than in April and October, and the values of *p*CO_2_ in June and August are higher than in April and October.

The temperature directly influences the production of methane by influencing the activity and structure of microflora^34^. In our study that temperature has no significant correlation with CH_4._ When the temperature rises, the dissolved oxygen concentration in the water decreases, which is more conducive to the production of methane. However, the concentration of methane in the Xilin River decreases with increasing temperature, which may be due to the significant increase in the activity of oxidizing bacteria in methane due to the increase in temperature, thus resulting in an increase in methane consumption.

Because of the strong correlation between flow rate and velocity, river sections with large flow rate usually have higher flow velocity. Higher velocity helps to increase the degree of surface turbulence and fragmentation, increase the area of contact between water and air, and accelerate the gas exchange rate at the water–air interface^35^. The flow velocity ranges from 0 to 1.2 m s^−1^ at all sampling points, which is a very low level, but the amount of carbon dioxide released from the Xilin River is considerable. Because of the large amount of carbon in the environment around the channel, it can be washed into the river network system by runoff or groundwater^9,36^.

The total alkalinity of the Xilin River is low, and there is less plankton in the water, which indicates that the river carbon dioxide mainly comes from terrestrial organic carbon rather than inorganic carbon. A large part of terrestrial organic carbon comes from net primary productivity.

The influence of nutrient on river CO_2_ affects the production and consumption of in situ CO_2_ in the river mainly through changing the balance of primary production and ecosystem respiration^36,37^. The Xilin River is a low-pollution region. Studies have shown that the increase of nutrient salt mainly promotes the growth of phytoplankton in the water, enhances photosynthesis, and then reduces the concentration of CO_2_ in the water due to carbon restriction in low-pollution rivers^38^.

### Influence of land use on GHG

The *p*CO_2_ value (> 10,000 ppm) in sand land is much higher than that of other land use types (< 10,000 ppm) due to groundwater recharge which had high CO_2_ content^39^ In large river systems, the *p*CO_2_ value in the groundwater system is approximately ten times higher than that in the surface water system. Except for the areas affected by human activities, the river alkalinity in sand land areas is the largest^40^. A large amount of carbon ions (HCO_3_^-^, CO_3_^2-^) and dissolved CO_2_ is released from groundwater recharge surface water under the action of photosynthesis and weathering^41^. The carbon ion reaction in surface water is released into the atmosphere, the variation range of subsurface temperature is small, and the dissolved CO_2_ exists stably in groundwater. However, after the groundwater recharge surface water is exposed to the river, the temperature in the atmosphere changes greatly, and the solubility of CO_2_ varies with the change of temperature^35^.

The Xilin River source area is a swamp area, and groundwater is one of the sources of river runoff in swamp area and is rich in dissolved carbon dioxide; a small amount of groundwater recharge thus also provides sufficient carbon dioxide for river water^39^. At the same time, the bog type in the Xilin River source area is low-lying bog, the initial stage of bog development with a low-lying surface, which often becomes the place where surface runoff and groundwater collect and pool. Water supply is mainly groundwater, and there are many minerals and nutrients in the water. The pH of water and peat is acidic to neutral. The results of the Xilin River source area are the same as the report where *p*CO_2_ was much higher than the downstream water^42^. The CH_4_ of swamp in the Xilin River is 8.14 mmol m^−2^ d^−1^, which is higher than the CH_4_ value of the Tibetan Plateau (4.19 mmol m^−2^ d^−1^)^43^. In the study of CH_4_ emissions of the Yukon River basin, both main stream and tributary showed that the upstream concentration of CH_4_ was lower than that of the midstream and downstream^38,44^. The emissions of CH_4_ from Xilin River also showed a similar distribution pattern.

In the Xilin River, the vegetation coverage in grassland is relatively high, similar to that in swamp. Carbon dioxide release from swamps coincides with the growth cycle of plants, and terrestrial organic carbon related to plants is thus one of the important sources of carbon dioxide in river water bodies^17^. Because of the low carbon density in the soil carbon pool, the soil carbon pool provides less carbon to the river carbon pool than the swamp. There are relatively few inundated-vegetation areas in grassland, and aquatic vegetation thus provides less organic carbon to rivers. On the other hand, the altitude of grassland cover areas is low, the water level of groundwater in mountain bodies is higher, and groundwater also acts as the source of river runoff^45^. The net primary productivity of the Xilin River Basin begins in April and reaches its peak in August, and the average annual net primary productivity from 1926 to 2017 is 185.38 gC m^−2^ a^−1^; to some extent, this shows that there is a strong relationship between river carbon and net primary productivity in the Xilin River Basin, and a part of the carbon dioxide in the river is provided by plant carbon. In the Xilin River Basin, the CH_4_ emission of grassland is 31.25 μmol m^−2^ d^−1^, and the CH_4_ emission of rivers is approximately 100 times that of grassland^46^.

### Influence of human activities on GHG

The CH_4_ of river water is mainly produced by methanogens in sedimentary layers after a series of fermentation processes in the anaerobic environment with acetate or CO_2_/H_2_ as a substrate^34^. An increase in temperature would stimulate the activity of soil/sediment methanogenic bacteria as well as promote a higher rate of organic matter degradation, which in turn would provide more substrates for methanogens to produce CH_4_^47,48^. The small Pond (Wolongquan) is mainly used for raising fish. Because of the large amount of breeding and artificial feed, it contains a large number of nutrients, which promotes the reproduction and growth of algae^49^. The growth process of plankton produces a large amount of fresh organic carbon, which stimulates the production of CH_4_. The proliferation of planktonic plants and animals leads to the reduction of oxygen concentration in the deep water layer, which creates an anaerobic environment for the production of CH_4_ and reduces CH_4_ oxidation^50^. The dissolved oxygen value is very low, which promotes the growth of anaerobes, and the river is in a eutrophication state, which produces more CH_4_ under the anoxic conditions^51,52^.

The *p*CO_2_ value of the Xilin Reservoir is 670.15 ± 114.48 ppm, which is higher than the background value of atmospheric carbon dioxide, and its *F*CO_2_ value (0.02 ± 0.02 mol m^−2^ d^−1^) is similar to that of an artificial lake. The construction of a reservoir changes the biogeochemical cycle of carbon and nitrogen in the basin; the flow rate slows down after the river enters the reservoir, which causes the deposition of plankton debris and other granular organic matter in the water. These changes will affect the production and release of greenhouse gas. Compared with rivers, reservoirs have longer hydraulic retention times, which is conducive to the accumulation of pollutants^53^. Phytoplankton debris, that is, endogenous organic matter, provides a rich source of easily degradable carbon for the accumulation of organic matter at the bottom of the reservoir and enhances the anaerobic conditions at the bottom of the reservoir, which thus provides a material basis and environmental conditions for the production of CH_4_. The flow velocity of water in the Xilin Reservoir is approximately zero, and there is no large fluctuation.

The Xilin River flows through an artificial lake in Xilinhot City, but the River flows intermittently to its downstream because of the impoundage of the Xilin Reservoir. The discharge amount of water in the Xilin Reservoir to the downstream is very small, which has little impact on *p*CO_2_ in the downstream. The artificial lake in Xilinhot City is composed of river water and reclaimed water. The artificial lake flow rate is close to zero, and its water–gas interface gas release rate is extremely low, and the *F*CO_2_ value is 0.02 ± 0.01 mol m^−2^ d^−1^. There is no obvious plankton in the lake, and reeds grow around the artificial lake. The *p*CO_2_ value in the artificial lake is higher than that in the atmosphere^54^. The CO_2_ produced by the respiration of aquatic plants inside the water body is basically the same as the CO_2_ fixed by the photosynthesis of aquatic plants.

The lower reaches of the Xilin River are located in the factory area and are greatly influenced by human activities, mainly by power stations and dairy farms; the power station extracts groundwater for production operations. After secondary treatment, sewage is discharged into the river and mixed with the Xilin River. The water discharged from the dairy farms also contains a large number of microorganisms, and the river contains a large number of organic substances as well as nitrogen and phosphorus compounds, which makes algae and microorganisms grow and reproduce; a large number of them exist in the river and promote river oxygen metabolism. The respiration of algae and microorganisms releases a small amount of carbon dioxide but microorganisms produce more CO_2_ and CH_4_ in the absence of oxygen^34^. The values of *p*CO_2_ (2,048.93 ± 660.43 ppm) and *F*CO_2_ (0.41 ± 0.74 mol m^−2^ d^−1^) in factory areas are higher than in other areas affected by human activities. The value of N_2_O in the factory area is higher, and the total nitrogen and total phosphorus in the river are positively correlated with the concentration of N_2_O^55^. The sewage discharged from the factory contains a large amount of nutrients, which makes the microbial activity produce a large amount of CH_4_ and N_2_O. With the discharge of sewage into the channel, the release of CH_4_ and N_2_O in the channel and downstream of the channel is indirectly affected^17^.

### Comparison with other rivers

Our estimated results for CO_2_ emissions in the Xilin River were greater than in most of the reported rivers, such as the Wuding River^56^, the Daning River^57^, and inland water in Africa^17^ (Table 3). The emissions of CH_4_ and N_2_O in the Xilin River were higher than the inland water in Africa. Because there are many land types in the Xilin River, the range of greenhouse gas emissions was larger than that of other rivers at home and abroad.

**Table 3:** Comparison of GHG emissions from river to atmosphere.

Description	River	Sites	*F*CO_2_ (mol m^−2^ d^−1^)	*F*CH_4_ (mmol m^−2^ d^−1^)	*F*N_2_O (μmol m^−2^ d^−1^)	References
Inland water	–	African	0.18–1.15	0.5–18.0	2.0–16.0	Borges et al.^17^
River	Wuding River	China	0.02–0.98	–	–	Ran et al.^56^
	Daning River	China	0.33 ± 0.47	–	–	Ni et al.^57^
	Xilin River	China	0.00–6.09	0.06–105.19	− 12.60–224.04	This study
Swamp	Phragmites marsh	China	0.01–1.83	0.48–9.60	–	Olsson et al.^58^
	Saltmarsh	Alabama	− 0.06 to − 0.03	0.00–6.00	–	Wilson et al.^59^
	Xilin River	China	0.13–3.36	1.88–30.38	− 3.05 to 25.62	This study
Pond	Crab-fish	China	–	0.11	–	Hu et al. (2016)
	Min River	China	0.01	129.06	–	Yang et al.^29^
	Xilin River	China	0.02–4.81	0.34–105.19	0.22–15.65	This study
Reservoir	Three Gorges	China	0.09–0.17	0.03–0.57	–	Zhao et al.^60^
	Shasta	America	0.03	0.69	–	Soumis et al.^53^
	Xilin River	China	0.00–0.05	0.06–0.56	0.17–8.34	This study

The carbon dioxide emissions from the swamp of the Xilin River were close to those of the Phragmites marsh, but the methane emissions were higher than those from the Phragmites marsh. Additionally, saltmarshes are sinks of carbon dioxide, and the values of methane emissions were between those of swamp and Phragmites marsh^58,59^. Due to the lower height of grassland vegetation, photosynthesis is stronger. The type of plant in the marsh area affects greenhouse gas emissions of water.

Carbon dioxide emissions were lower for the Min River than for the Xilin River, but the methane emissions of the Min River were higher than those of the Xilin River. The emissions of CH_4_ of Min river was higher than the Xilin river, because the drainage of the Wolongquan pond was lower than the Min river. The drainage of the Min river significantly enhance CH_4_ emissions^29^. The Wolongquan pond of the Xilin River is mainly used for farming fish with great artificial intervention, which proves that the addition of nutrients has a great influence on the greenhouse gas emissions of water bodies.

In the Xilin river reservoir, the emissions of CO_2_ and CH_4_ were equal to the Three Gorges and Shasta reservoir^53,60^. For reservoir, the flow velocity of surface water is slow, which causes the emissions of greenhouse were less. And the deep water created well-oxygenated conditions, resulting in lower methane emissions.

## Conclusions

In this study we estimated emissions of greenhouse gas from the Xilin River, which is characterized by different land-use types and various degrees of human impacts. The results showed that the hydrological drainage network of the Xilin River was oversaturated in GHG (CO_2_, CH_4_ and N_2_O) with respect to the atmospheric concentrations. For the hydrosystem of the Xilin River Basin, CO_2_ emissions accounted for 63.35% of the three GHG emissions, whereas CH_4_ and N_2_O emissions accounted for 35.98% and 0.66%, respectively. GHG emissions from the Xilin river were dominated by CO_2_ emissions and were interpreted as being supplied by terrestrial carbon transportation and groundwater replenishment and by wastewater discharges. In future work, sampling should cover more sites with a greater frequency to better quantify the magnitude of CO_2_, CH_4_ and N_2_O emissions at diurnal and monthly scales before upscaling them to annual estimates. Comparing the differences in greenhouse gas emissions after the cut-off, it is possible to predict the total greenhouse gas emissions after global river drying.
